# Antiviral prophylaxis inhibits cytomegalovirus reactivation in critical illness

**DOI:** 10.1186/cc14195

**Published:** 2015-03-16

**Authors:** NJ Cowley, A Owen, J Millar, SC Shiels, RL Woolley, NJ Ives, H Osman, P Moss, JF Bion

**Affiliations:** 1 University Hospital Birmingham, UK; 2 University of Birmingham, UK

## Introduction

Reactivation of latent cytomegalovirus (CMV) can lead to viraemia or CMV disease and has been detected in up to 30% of critically ill patients without prior history of immune suppression. However, the clinical importance of this observation remains unclear. We report a proof-of-concept randomised controlled trial of two antiviral drugs in intensive care patients to determine their impact on CMV reactivation.

## Methods

We conducted a single-centre randomised controlled study of high-dose valaciclovir or low-dose valganciclovir prophylaxis, as compared with standard care, in CMV seropositive patients in the ICU at Queen Elizabeth Hospital Birmingham, UK. Patients were excluded if CMV seronegative. Study participants randomised to a study drug received either 450 mg valganciclovir daily enterally (or ganciclovir intravenously) or 2 g valaciclovir four times daily enterally (or aciclovir intravenously) for a period of up to 28 days. Blood was collected for CMV viral load during the 28-day study period. The primary outcome measure was reactivation of CMV in blood above 20 copies.ml^-1^ (assay detection limit) by day 28.

## Results

**Figure 1:**
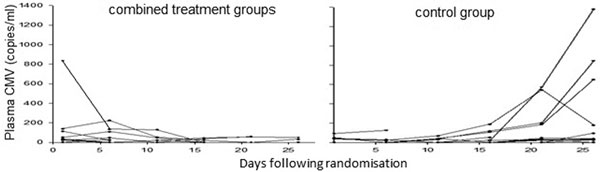
**CMV reactivation over time**. Each line represents a single patient.

A total of 124 patients were randomised; 44 control, 34 valaciclovir, and 46 valganciclovir. Recruitment to the valaciclovir arm was halted early because of an imbalance in mortality (44% mortality vs. 19% in other arms). Independent blinded review of all deaths did not reveal any deaths attributable to unexpected causes. Fourteen patients were excluded from the primary analysis because of baseline CMV reactivation. CMV reactivation occurred in 30% (12/40) of the control arm but only 3% (1/39) in the valganciclovir arm (RR: 0.09 (95% CI: 0.01, 0.6)). When the two treatment arms were considered together, reactivation was observed in only 4% (3/70) (RR: 0.1 (95% CI: 0.04, 0.5)). See Figure 1.

## Conclusion

This is the first study in critical care to assess the feasibility of antiviral prophylaxis to prevent CMV reactivation in a mixed population of critically ill patients. Low-dose valganciclovir was shown to suppress CMV reactivation as effectively as higher-dose valaciclovir.

## Acknowledgements

Research funded by the NIHR under the RfPB Programme (PG 1010 23225). Views expressed are of the authors and not necessarily the NHS, NIHR, or DOH.

